# Metabolomics Analysis of Hippocampus and Cortex in a Rat Model of Traumatic Brain Injury in the Subacute Phase

**DOI:** 10.3389/fnins.2020.00876

**Published:** 2020-09-04

**Authors:** Fei Zheng, Yan-Tao Zhou, Peng-Fei Li, En Hu, Teng Li, Tao Tang, Jie-Kun Luo, Wei Zhang, Chang-Song Ding, Yang Wang

**Affiliations:** ^1^College of Electrical and Information Engineering, Hunan University, Changsha, China; ^2^Laboratory of Ethnopharmacology, Institute of Integrated Traditional Chinese and Western Medicine, Xiangya Hospital, Central South University, Changsha, China; ^3^College of Integrated Traditional Chinese and Western Medicine, Hunan University of Chinese Medicine, Changsha, China; ^4^School of Informatics, Hunan University of Chinese Medicine, Changsha, China

**Keywords:** traumatic brain injury, hippocampus and cortex, metabolomics, LC-MS/MS, subacute phase

## Abstract

Traumatic brain injury (TBI) is a complex and serious disease as its multifaceted pathophysiological mechanisms remain vague. The molecular changes of hippocampal and cortical dysfunction in the process of TBI are poorly understood, especially their chronic effects on metabolic profiles. Here we utilize metabolomics-based liquid chromatography coupled with tandem mass spectrometry coupled with bioinformatics method to assess the perturbation of brain metabolism in rat hippocampus and cortex on day 7. The results revealed a signature panel which consisted of 13 identified metabolites to facilitate targeted interventions for subacute TBI discrimination. Purine metabolism change in cortical tissue and taurine and hypotaurine metabolism change in hippocampal tissue were detected. Furthermore, the associations between the metabolite markers and the perturbed pathways were analyzed based on databases: 64 enzyme and one pathway were evolved in TBI. The findings represented significant profiling changes and provided unique metabolite–protein information in a rat model of TBI following the subacute phase. This study may inspire scientists and doctors to further their studies and provide potential therapy targets for clinical interventions.

## Introduction

Traumatic brain injury (TBI) is defined as brain tissue damage caused by a mechanical force. This worldwide health problem causes high mortality and disability in all ages and countries. It has been estimated that the cases afflicted by TBI exceed 50 million and cost 400 billion US dollars annually (Maas et al., 2017). Unfortunately, specific TBI treatments failed because of very complicated pathophysiology (Berger et al., 2007; Zetterberg et al., 2013; Lichtman et al., 2014). This motivates studies to identify the possible potential pathogenic mechanisms of TBI in search for the underlying targets in brain sites.

To overcome difficulties in the exploration of pathogenic mechanisms following TBI (Maas et al., 1999), omics approaches including transcriptomics, proteomics, and metabolomics have become an emerging technology to discover biomarkers and biological processes by using a global untargeted approach (Lommen et al., 2007). Of omics platforms, metabolomics has become a powerful tool with the aid of quantifiable systems biology research in high-throughput metabolic data sets (Vasilopoulou et al., 2016). This method has been carried out for non-targeted metabolic profiling to reveal novel biomarkers and biochemical pathways to gain new insights into the potential pathogenic mechanisms of TBI (Zhang et al., 2010; Feala et al., 2013; Wolahan et al., 2015). Previous studies showed that the metabolomic differences induced by TBI were distinguished (Chitturi et al., 2018; Dickens et al., 2018; McGuire et al., 2019), and the metabolic signatures and progressions might be beneficial to clinical TBI diagnosis or therapeutic possibilities (Chitturi et al., 2018; Dickens et al., 2018; McGuire et al., 2019).

The characteristics of metabolism profiles during TBI refer to neurologic function alteration and cognitive impairment (Werner and Engelhard, 2007; Chauhan, 2014; Barkhoudarian et al., 2016). Moreover, evidence suggests that disorders (including glutamate excitotoxicity, neuroplasticity, metabolic disruption, and mitochondrial dysfunction) existed for long-lasting periods after TBI (Maas et al., 2008; Faden, 2011; Turner et al., 2013). The hippocampus and the cortex especially show significant aberrations in neural pathways that are associated with spatial learning memory and executive function (Boone et al., 2017). Therefore, investigating the pathogenesis of the hippocampus and the cortex may provide useful information about the potential mechanisms. Previous studies have obtained metabolic fingerprints of the cortex and the hippocampus of TBI, including energy metabolism disorder, oxidative stress, excitotoxic damage, and neuronal damage (Girgis et al., 2016). A non-targeted nuclear magnetic resonance (NMR) metabolomics study examined their metabolic profiles in five brain regions of chronic post-TBI injury (Viant et al., 2005). Although cortical and hippocampal metabolomics studies of acute and chronic TBI were well documented (Pierce et al., 1998; Casey et al., 2008; McGuire et al., 2019), we believe that a description on metabolomics of subacute TBI remains urgent. Because as a transition period, the subacute phase-induced metabolic disturbance may last long, and the pathological cascades may determine the prognosis and the development of TBI.

**FIGURE 1:**
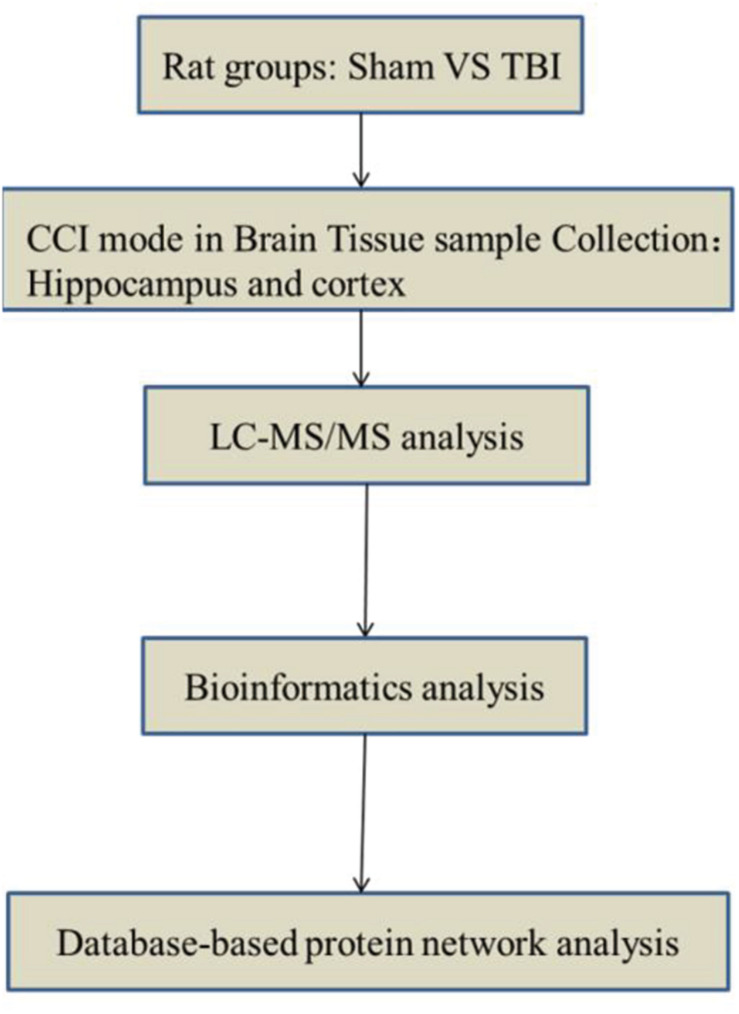
Flow chart of the study design.

In this study, we focused on the metabolic changes of subacute TBI in a rat model. Alterations in brain metabolism in the cortex and the hippocampus after subacute TBI in rats were observed and analyzed by non-targeted liquid chromatography coupled with tandem mass spectrometry (LC-MS/MS) metabolomics method. The results tend to provide a more comprehensive understanding of TBI pathophysiology and the therapeutic strategies in the subacute phase. The workflow is illustrated in Figure 1.

## Materials and Methods

### Ethical Approval

All animal experiments were performed according to the relevant guidelines for animal research established by Xiangya Hospital Central South University. All procedures involved in the experiments were approved in compliance with the regulations of the Medical Ethics Committee at Xiangya Hospital Central South University.

### CCI Model in Rats

Forty male Sprague–Dawley rats (weight: 200–250 g) were obtained from the Laboratory Animal Research Center of Central South University. All rats were between 6 and 8 weeks of age. They were bred with free access to food and water for at least 1 week and housed with 12-h circulation. The rats were randomly divided into two groups: (1) sham group (*n* = 10): operated rats that underwent the surgical procedures except for an impact-experienced trauma and (2) TBI group (*n* = 10): rats that experienced controlled cortical impact (CCI). The CCI model was performed following a previous description (Xing et al., 2016). Each rat was administered with 3% pentobarbital (50 mg/kg) prior to surgery. A craniotomy (5 mm in diameter), located at the center of the bregma and the lambda suture lines, was performed with a dental drill at the right portion of the skull. The hammer was made of an automated, controlled-pneumatic-impact device (PSI TBI-0310 Impactor, Precision Systems & Instrumentation, Fairfax Station, VA, United States). The parameters of the brain injury consisted of a 6.0 m/s impact velocity, 5 mm impact depth, and 500 ms dwell time. Then, the incision was closed with sutures and the animal was placed on a heating cushion to maintain the normal core temperature after the impact.

### Brain Tissue Sample Collection

On the 7th day after CCI, the animals were deeply injected with intraperitoneal anesthetic pentobarbital and then perfused with 200 ml of 0.9% normal ice-cold saline. The hippocampal and the cortical tissues surrounding the injured side were rapidly dissected and placed in cryopreservation tubes. Then, the samples were stored in liquid nitrogen and stored at −80°C until LC-MS/MS analysis.

### Modified Neurological Severity Score

To assess the neurological functions, we used the modified Neurological Severity Score (mNSS) to evaluate the neurological impairments of CCI rats on the 7th day (Xing et al., 2016). The mNSS includes 18 points comprising motor (six points), sensory (two points), beam balance (six points), absent reflexes, and abnormal movements (four points). One point refers to the failure of one task; no points are given for success. Higher scores suggest more severity in such rats (normal score: 0, maximal deficit score: 18).

### LC-MS/MS Analysis

The LC-MS/MS method was done for quantitative analysis. Mobile phase A (methanol) was comprised of 25 mM ammonium acetate and 25 mM ammonium hydroxide with water. Mobile phase B (Acetonitrile) was applied at a flow rate of 0.4 ml/min by using a 4 l injection volume. Generic high-performance liquid chromatography (HPLC) gradient was used to determine the metabolites in terms of time (0.0, 1.0, 11.0, 14.0, 16.5, 18.5, 20.5, 25.0, 25.1, and 34.0 min): A (10, 10, 13, 20, 30, 50, 80, 80, 10, and 10%) and B (90, 90, 87, 80, 70, 50, 20, 20, 90, and 90%). Agilent 1260 Infinity HPLC (Agilent J&W Scientific, Folsom, CA, United States) with a Waters amide column (2.1 × 100 mm, 3.5 μm) was carried out for the HPLC analysis.

Subsequently, MS/MS analysis was carried out on the Q-Exactive MS/MS (Thermo) in both positive and negative ion modes. The MS parameters for the probe were set based on optimized conditions (auxiliary gas: 13, sheath gas: 40, aux gas heater temperature: 400°C, capillary temperature: 350°C, S-lens: 55, spray voltage: 3.5 kV for positive mode and negative mode). Data-dependent acquisition method was built based on the following parameters: full scan range: 60–900 m/z; normalized collision energies: 10, 17, 25 or 30, 40, 50; automatic gain control MS1: 3e6; ddMS2: 2e5; isolation window: 1.6 m/z; resolution MS1: 70,000; ddMS2: 17,500; maximum injection time MS1: 100 ms; and ddMS2: 45 ms. The full-scan method was set as follows: full scan range: 60–900 m/z, automatic gain control: 3 × 10^6^ ions, resolution: 140,000, and maximum injection time: 100 ms.

### Data Analysis and Statistics

Data (Supplementary Material 1, 2) were analyzed by multivariate statistical analysis, including principal component analysis (PCA), partial least-square discrimination analysis (PLS-DA), and orthogonal partial least-square discriminant analysis (OPLS-DA) using SIMCA-P software (version 11.0; Umetrics AB, Umea, Sweden) with log10 transferred and pareto-scale scaling. A PCA model of data set was visualized by clustering trend. PLS-DA and OPLS-DA models were employed for predicted probabilities and supervised discrimination. The values of R2X and Q2Y as goodness-of-fit were performed to estimate how accurate is the validated model and to describe the predictive ability. A total of 100 iterations of the permutation test in the PLS-DA model was subsequently used for reliability validation. According to the OPLS-DA analysis, the potentially different metabolites were selected by variable importance in the projection (VIP) values (VIP>1). Additionally, the potential metabolic signatures with false discovery rate (FDR) ≤ 0.05 and fold change (FC) > 1.5 or < 0.67 were identified. To illustrate the relevant pathways enriched by metabolites, different identifications were investigated using Kyoto Encyclopedia of Genes and Genomes (KEGG) in Metaboanalyst 3.0^1^ with *P* < 0.05.

We identified the metabolites by mzCloud and ChemSpider. The exacted mass of each feature was submitted to ChemSpider with selected databases of BioCyc, Human Metabolome Database, KEGG, and LipidMAPS. The relative standard deviation of the different metabolites was calculated. We extracted the proteins which are related to significantly different metabolites *via* the Human Metabolome Database (HMDB). Then, we collected conserved gene-based proteins among these proteins *via* BLAST-NCBI^2^ per protein whose mRNA identification >80% was selected. Furthermore, we searched for traumatic brain injury-associated proteins *via* GeneCard. Finally, we analyzed metabolite–protein associations through the KEGG pathway. The biological pathway, biological process, and their correlation networks were drawn by Gingo package in cytoscape (version 3.5) software based on the KEGG pathway database.

Data for the mNSS tests were expressed as mean ± SD. Prism Graph Pad (version 8.0) software was used for statistical analysis and graphing. Statistical differences were determined using Student’s *t*-test with *P* set to <0.05 considered as statistically significant.

## Results

### Neurological Deficit Assessments

**FIGURE 2:**
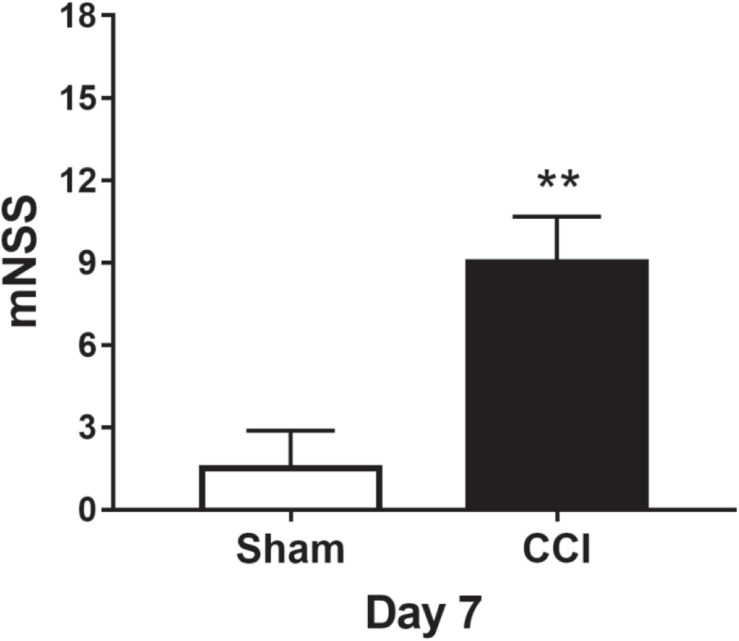
The assessment of neurological deficits on day 7. **p < 0.01 vs. sham.

Before the surgical operation, the rats were evaluated by mNSS measurement, and each rat had 0 score. On the 7th day after the operation, the mNSS scores of the sham group were less than three points. When compared with the sham group, the TBI group showed significantly higher scores (*p* < 0.01, more than nine points). The results indicated that TBI led to marked neurological impairments (Figure 2).

### Metabolomic Fingerprints to Distinguish TBI Disease States in the Cortex and the Hippocampus

**FIGURE 3:**
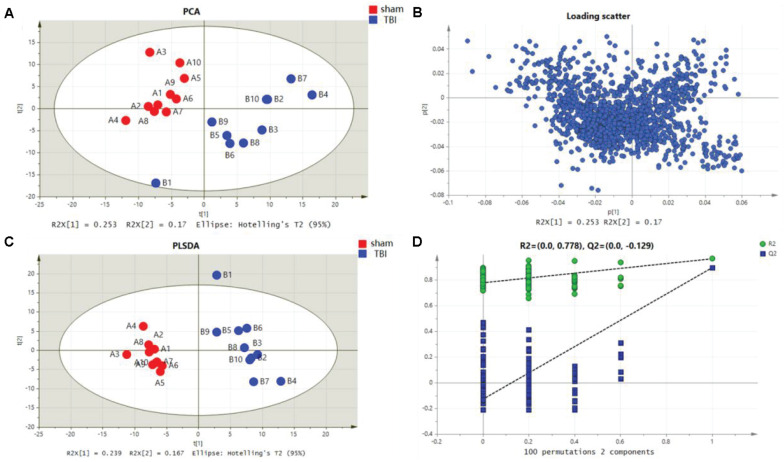
Metabolomic fingerprint analysis for the cortex in principal component analysis (PCA) and partial least-square discrimination analysis (PLS-DA). **(A)** PCA plots in cortical traumatic brain injury (TBI) vs. sham. **(B)** Loading plot corresponding to the PCA score scatter plot for cortical TBI. **(C)** PLS-DA results for cortical TBI [R2Y (cum) = 0.965 and Q2Y (cum) = 0.895]. **(D)** Validation plot obtained from 100 permutation tests for the cortex.

**FIGURE 4:**
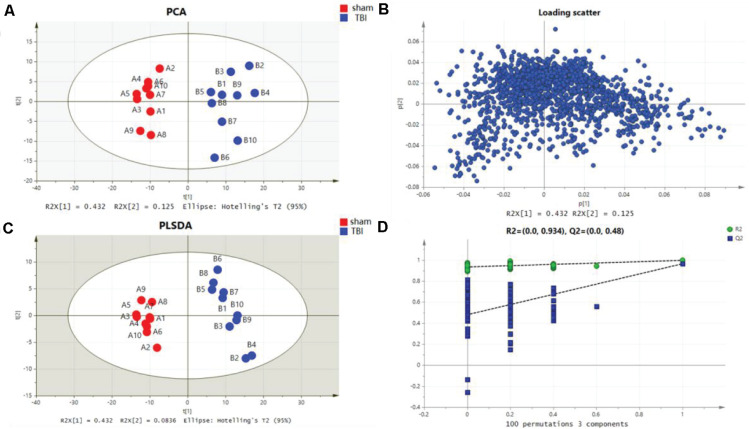
Metabolomic fingerprint analysis for the hippocampus in principal component analysis (PCA) and partial least-square discrimination analysis (PLS-DA). **(A)** PCA plots in hippocampal traumatic brain injury (TBI) versus sham. **(B)** Loading plot corresponding to the PCA score scatter plot for hippocampal TBI. **(C)** PLS-DA results for hippocampal TBI [R2Y (cum) = 0.997 and Q2Y (cum) = 0.964]. **(D)** Validation plot obtained from 100 permutation tests for the hippocampus.

Metabolomic profiling was performed on the 7th day after the operation. The metabolic features in the cortex and the hippocampus were detected. A PCA indicated that the TBI-induced cortex or hippocampus tissue group was clearly distinguished from the sham group. The first two principal components in their PCA score plot showed a clear classification between the TBI and the sham groups (Figures 3A, 4A). The PCA loading scatter profiled differences in the direction of the first predictive principal component during the progress of TBI as the most distinct separation features (Figures 3B, 4B). Furthermore, to minimize the effects between groups, we embedded a group separation trend for the TBI and the sham groups into the PLS-DA model. The PLS-DA model with R2Y and Q2Y [R2Y (cum) = 0.965 and Q2Y (cum) = 0.895 for the cortex and R2Y (cum) = 0.997 and Q2Y (cum) = 0.964 for the hippocampus] was obtained to indicate perfect fitting and reliable prediction (Figures 3C, 4C). In addition, a repeated permutation test (consisting of 100 random permutation texts) validated the model without overfitting (R2 was 0.778 for the cortex and 0.934 for the hippocampus; Q2 was −0.129 for the cortex and 0.48 for the hippocampus) (Figures 3D, 4D).

### Selection and Identification of Characteristic Metabolites in the Cortex and the Hippocampus

**FIGURE 5:**
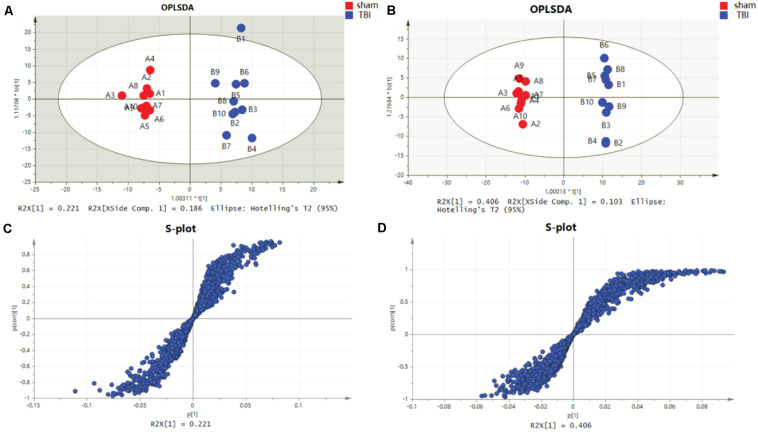
Orthogonal partial least-squares discriminant analysis (OPLS-DA) mode and S-plot of the subacute traumatic brain injury (TBI) vs. sham group. **(A)** OPLS-DA models for cortical TBI vs. sham. **(B)** OPLS-DA models for TBI of the hippocampus vs. sham. **(C)** OPLS-DA model for TBI of the cortex shown in the S-plot. **(D)** OPLS-DA model for TBI of the hippocampus is shown in the S-plot, which illustrates the relationship between correlation and covariance.

The characteristic metabolites related to TBI-induced cortical or hippocampal tissues were detected by OPLS-DA. In the OPLS-DA plot, the reliable potential signatures were visible by maximizing the metabolite pattern between the TBI and the sham groups (Figures 5A,B). Potential metabolites were filtered by selecting the variable projected in an OPLS-DA model with high contributions according to the values of VIP shown in S-plot (Figures 5C,D). The potential metabolic results are summarized in Tables 1, 2. The characteristic metabolites were considered significant according to the following processes: (1) metabolites with VIP > 1, (2) Student’s *t*-test with *P*-value < 0.05, (3) FDR < 0.05, and (4) FC > 1.5 or < 0.67. Among these metabolites, eight metabolites for the cortex (betaine, trigonelline, palmitoylcarnitine, uric acid, guanidinoethyl sulfonate, ecgonine, 2′-deoxyinosine, and cholecalciferol) and seven metabolites for the hippocampus (L-norleucine, adenine, ascorbic acid, hypotaurine, propionylcarnitine, betaine, and palmitoylcarnitine) were selected for further bioinformatic analysis.

**TABLE 1:** List of significant metabolites identified from the orthogonal partial least-squares discriminant analysis (OPLS-DA) mode for cortical traumatic brain injury (TBI).

Name	MW	RT (min)	KEGGID	VIP^*a*^	*p*-value^*b*^	False discovery rate (FDR)^*c*^	FDR^*d*^	FC
L-Phenylalanine	165.08	5.99	C00079	1.41	2.23E-05	2.23E-05	2.26E-05	0.68
DL-Carnitine	161.10	15.94	C00318	1.15	2.40E-03	2.40E-03	2.40E-03	0.73
L-Norleucine	131.09	6.84	C01933	1.19	3.08E-04	3.08E-04	3.08E-04	0.74
Betaine	117.08	6.14	C00719	2.02	1.55E-07	1.55E-07	1.55E-07	0.49
Adenine	135.05	2.13	C00147	1.16	1.47E-02	1.47E-02	1.47E-02	1.49
Proline	115.06	9.10	C00148	1.32	4.24E-05	4.24E-05	4.25E-05	0.70
L-(-)-Methionine	149.05	7.95	C00073	1.34	1.45E-04	1.45E-04	1.45E-04	0.68
Trigonelline	137.05	7.27	C01004	1.88	2.77E-03	2.77E-03	2.77E-03	0.51
Valine	117.08	8.87	C00183	1.15	4.01E-04	4.01E-04	4.01E-04	0.75
DL-Tryptophan	204.09	6.66	C00525	1.21	3.30E-04	3.30E-04	3.30E-04	0.73
Palmitoylcarnitine	399.33	1.99	C02990	3.08	1.52E-07	1.52E-07	1.55E-07	0.21
Pipecolic acid	129.08	22.10	C00408	1.25	7.79E-04	7.79E-04	7.79E-04	0.70
N,N-Dimethylsphingosine	327.31	0.91	C13914	1.27	2.65E-03	2.65E-03	2.65E-03	0.68
Uric acid	168.03	11.47	C00366	3.02	2.46E-06	2.46E-06	2.47E-06	0.22
Guanidinoethyl sulfonate	167.04	8.28	C01959	1.55	5.55E-04	5.55E-04	5.55E-04	0.60
Ecgonine	185.10	2.16	C10858	2.47	2.12E-05	2.12E-05	2.13E-05	3.00
2′-Deoxyinosine	252.09	2.54	C05512	1.36	3.84E-03	3.84E-03	3.84E-03	0.64
Cholecalciferol	384.34	0.88	C05443	1.41	1.67E-02	1.67E-02	1.67E-02	2.01

**^*a*^Variable importance in the projection (VIP) was obtained based on OPLS-DA with a value > 1.0. ^*b*^The p-values were calculated using two-tailed Student’s t-tests. ^*c*^FDR was calculated using the Benjamini–Hochberg method. Metabolites with FDR values ≤ 0.05 were considered as significant. ^*d*^Fold change was calculated based on the arithmetic mean value of each group. Metabolites were obtained when the fold change was of a value more than 1.5 and a value less than 0.66.**

**TABLE 2:** List of significant metabolites identified from the orthogonal partial least-squares discriminant analysis (OPLS-DA) mode for the hippocampus.

Name	MW	RT (min)	KEGGID	VIP^*a*^	*p*-value^*b*^	FDR^*c*^	FC^*d*^
Palmitoylcarnitine	399.33	2.02	C02990	1.41	1.68E-07	1.68E-07	3.20
Betaine	117.08	6.17	C00719	1.24	9.74E-07	9.74E-07	1.72
L-Norleucine	131.09	6.07	C01933	1.05	4.40E-03	4.40E-03	1.72
Adenine	135.05	2.14	C00147	1.41	3.11E-05	3.11E-05	0.45
Ascorbic acid	176.03	1.75	C00072	1.41	6.37E-05	6.37E-05	2.26
Hypotaurine	109.02	13.65	C00519	1.25	4.20E-05	4.20E-05	1.87
Propionylcarnitine	217.13	8.27	C03017	1.61	8.14E-04	8.14E-04	2.83

**^*a*^Variable importance in the projection (VIP) was obtained based on OPLS-DA with a value > 1.0. ^*b*^The p-values were calculated using two-tailed Student’s t-tests. ^*c*^FDR was calculated using the Benjamini–Hochberg method. Metabolites with FDR values ≤ 0.05 were considered as significant. ^*d*^Fold change was calculated based on the arithmetic mean value of each group. Metabolites were obtained when the fold change was of a value more than 1.5 and a value less than 0.66.**

**FIGURE 6:**
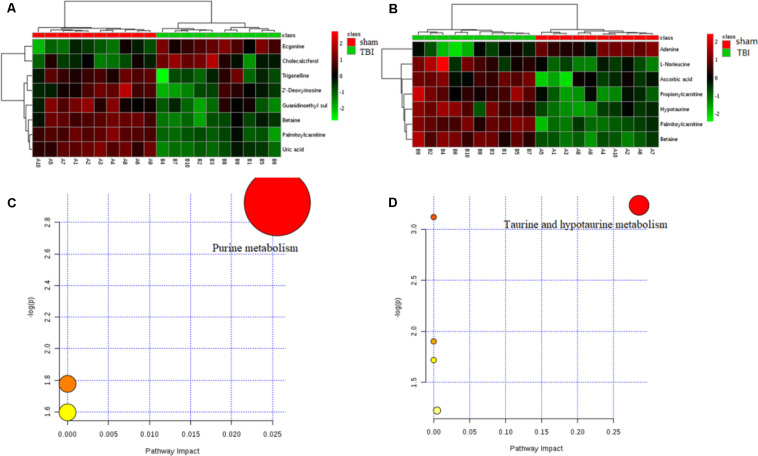
Results of heat maps and enrichment analysis of pathways. In the heat maps, the green color represents downregulation and the red color represents upregulation. For a metabolome view of the pathways, the colors of the nodes are determined based on the *P*-value of the pathway, and the radii are based on the impact values of their pathways. **(A)** Heat map of different metabolites in traumatic brain injury (TBI) of the cortex. **(B)** Heat map of different metabolites in TBI of the hippocampus. **(C)** Disturbed metabolic pathway associated with TBI of the cortex on day 7. **(D)** Disturbed metabolic pathway associated with TBI of the cortex on day 7.

**FIGURE 7:**
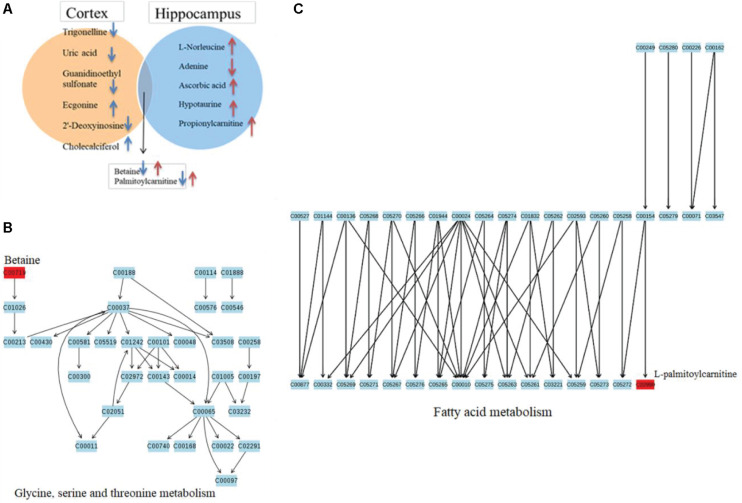
Venn diagram describing the different VIP metabolites in two distinctive brain tissues after traumatic brain injury (TBI) on day 7. **(A)** A Venn diagram indicates the different metabolites corresponding to two brain tissues, showing with blue arrows the upregulated or downregulated cortical metabolites and red ones for those of the hippocampus. The overlap display shows that the two metabolites are both in response to subacute TBI in the two brain tissues, with different trends. **(B)** Pathway topology analysis associated with betaine in glycine, serine, and threonine metabolism was carried out by a metaboanalyst. **(C)** Pathway topology analysis associated with palmitoylcarnitine in fatty acid metabolism was carried out by a metaboanalyst.

Of these metabolites, six metabolites in the cortex of the TBI group were downregulated in comparison with those of the sham group, and six other metabolites were decreased in the hippocampus. These metabolic profiles, shown in heat maps, suggested a decreased alteration in the metabolic tissue of TBI during the subacute phase (Figures 6A,B). A pathway analysis by a metaboanalyst revealed significantly different metabolites in the cortex or the hippocampus. As shown in Figures 6C,D, the identified metabolites were involved in purine metabolism for the cortical tissue and in taurine and hypotaurine metabolism for the hippocampal tissue, respectively. The results of Venn graphics (Figure 7) showed 13 metabolites in two categories for the cortex and the hippocampus. The areas of overlap in the Venn graphics suggested that betaine and palmitoylcarnitine were both detected in the cortex and the hippocampus, but they changed in opposite trends. Moreover, betaine and palmitoylcarnitine were significantly enriched in glycine serine and threonine metabolism and fatty acid metabolism through analysis with a metaboanalyst following the subacute TBI. The results demonstrated that these metabolite alterations may characterize the variable metabolomics response to subacute TBI.

### Construction of Metabolite-Correlated Protein Network Based on Database

**FIGURE 8:**
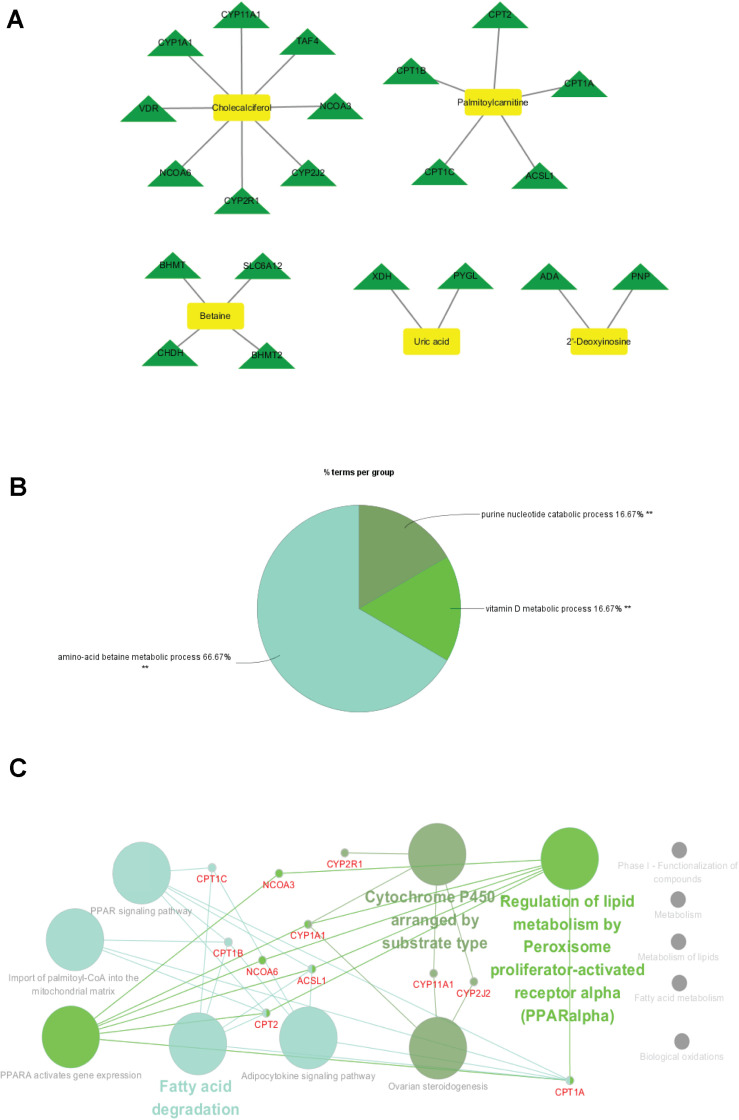
Metabolite-related proteins identified from the database and their associated analysis were visualized by the Cytoscape tool. **(A)** Representative metabolite–protein interaction network of the cortex, extracted from the database during the subacute phase. The network was constructed with metabolite compounds (yellow and rectangle) and protein enzymes (green and triangle) as nodes. **(B)** A pie chart provides a visualization of the three biological processes enriched by the proteins. **(C)** The protein pathway network provides a view of the functional relationships among proteins.

**FIGURE 9:**
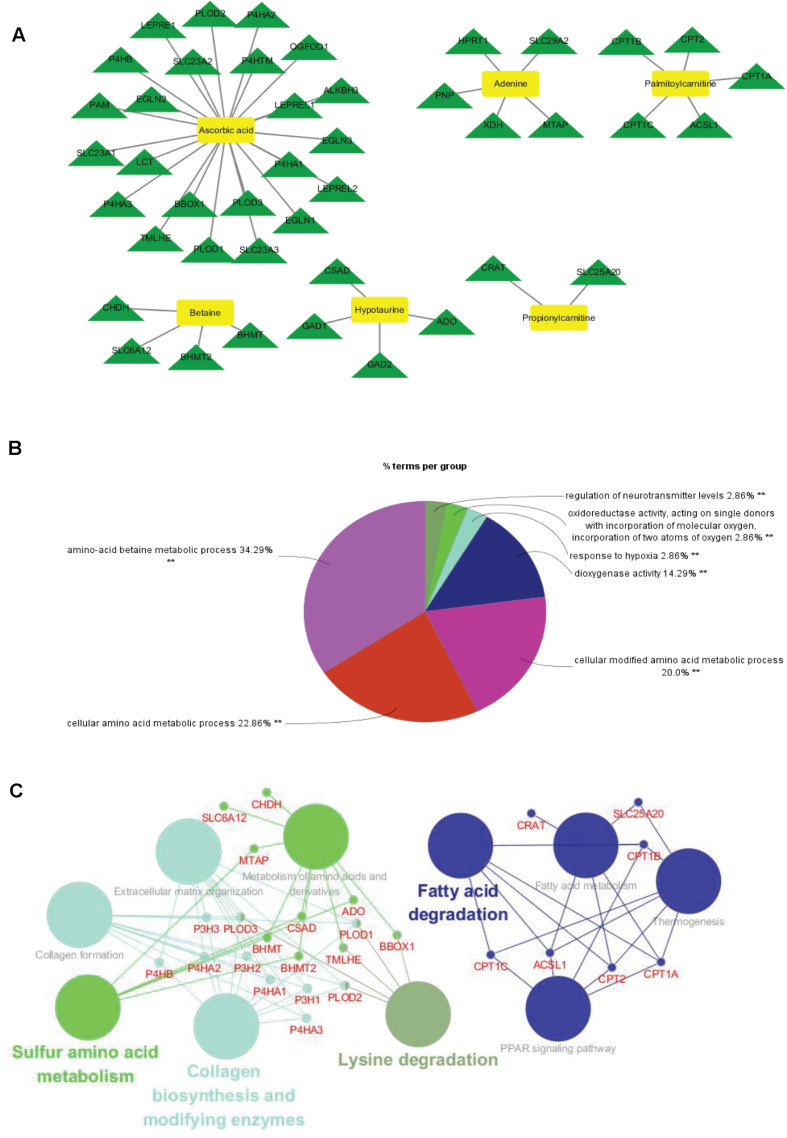
Metabolite-related proteins identified from the database and their associated analysis were visualized by the Cytoscape tool. **(A)** Representative metabolite–protein interaction network of the hippocampus, extracted from the database following the subacute phase. The network was constructed with metabolite compounds (yellow and rectangle) and protein enzymes (green and triangle) as nodes. **(B)** A pie chart provides a visualization of the seven biological processes enriched by the proteins. **(C)** The protein pathway network provides a view of the functional relationships among proteins.

To identify the metabolite-related proteins in the cortex and the hippocampus at the subacute period, we employed a metabolite–protein association network based on HMDB analysis (Figures 8, 9). Tables 3, 4 showed that a total of 21 proteins relevant to eight metabolites for the cortex and 43 proteins for seven metabolites for the hippocampus were extracted from the HMDB. For the underlying mechanism of the metabolite-related proteins, 64 proteins were analyzed to elucidate the biological processes and pathways by Cytoscape.

**TABLE 3:** Different metabolites in cortical traumatic brain injury and related proteins revealed by the Human Metabolome Database analysis.

Name	KEGGID	HMDB ID	Court of proteins
Betaine	C00719	HMDB0000043	4 enzymes
Trigonelline	C01004	HMDB0000875	–
Palmitoylcarnitine	C02990	HMDB0000222	5 enzymes
Uric acid	C00366	HMDB0000289	2 enzymes
Guanidinoethyl sulfonate	C01959	HMDB0003584	–
Ecgonine	C10858	HMDB0006548	–
2′-Deoxyinosine	C05512	HMDB0000071	2 enzymes
Cholecalciferol	C05443	HMDB0014315	8 enzymes

**TABLE 4:** Different metabolites in hippocampal traumatic brain injury and related proteins revealed by the Human Metabolome Database analysis.

Name	KEGGID	HMDB ID	Court of proteins
Palmitoylcarnitine	C02990	HMDB0000222	5 enzymes
Betaine	C00719	HMDB0000043	4 enzymes
L-Norleucine	C01933	HMDB0001645	–
Adenine	C00147	HMDB0000034	5 enzymes
Ascorbic acid	C00072	HMDB0000044	23 enzymes
Hypotaurine	C00519	HMDB0000965	4 enzymes
Propionylcarnitine	C03017	HMDB0000824	2 enzymes

Within the classification of the pie chart (Figures 8B, 9B), three biological processes related to 21 proteins and their related metabolites in the cortex on day 7, including amino acid betaine metabolic process followed by purine nucleotide catabolic process, and seven classifications of biological processes were mainly involved in amino acid betaine metabolic process and cellular amino acid metabolic process for the hippocampus. Moreover, to gain protein profiling, they were imported into Cytoscape for visualization of their corresponding enriched pathways by mapping these proteins into the KEGG reference. The relationship networks with their pathway associations were built by Cytoscape (Figures 8C, 9C). Our results revealed that 21 related proteins were involved in the two main series of pathways including lipid metabolism (fatty acid degradation) and endocrine system (PPAR signaling pathway, adipocytokine signaling pathway, and ovarian steroidogenesis) in the cortex on day 7, and there were four main series of pathways including lipid metabolism (fatty acid degradation and fatty acid metabolism), environmental adaptation (thermogenesis), endocrine system (PPAR signaling pathway), and amino acid metabolism (lysine degradation). The findings indicated that the 43 related proteins were involved in hippocampal TBI on day 7.

### Construction of TBI-Related Protein Network Based on Database

**FIGURE 10:**
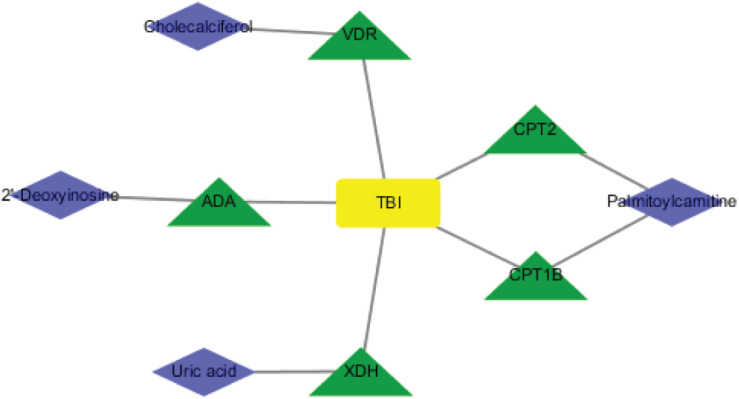
Perspective of traumatic brain injury (TBI) perturbation on integrated proteins, cortical metabolites: a network of TBI-causing candidate proteins (green and triangle) and cortical metabolites (blue and diamond).

**FIGURE 11:**
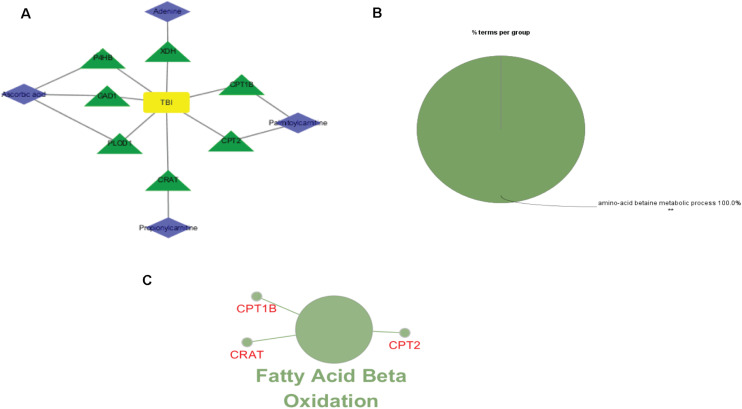
Perspective of traumatic brain injury (TBI) perturbation on integrated proteins, hippocampal metabolites, and pathway: **(A)** network of TBI-causing candidate proteins (green and triangle) and hippocampal metabolites (blue and diamond). **(B)** Gene Ontology analysis of seven hippocampal metabolite-related proteins. **(C)** A subnetwork of three proteins enriched in fatty acid beta oxidation.

**FIGURE 12:**
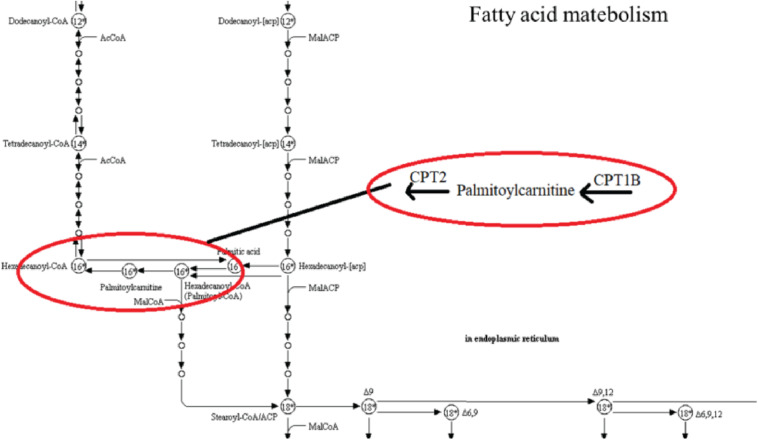
Kyoto Encyclopedia of Genes and Genomes (KEGG) pathway from an original KEGG pathway. Fatty acid metabolism KEGG pathway shows the interactions of proteins and metabolites with red circles.

To investigate the underlying pathological mechanisms of TBI, 12 TBI-related proteins from 64 metabolite-associated proteins were performed based on GeneCards database. The results showed TBI-induced protein distribution networks with five proteins and their related four metabolites for the cortex and seven proteins and their related four metabolites for the hippocampus, respectively (Figures 10, 11A). In addition, we performed a pathway enrichment analysis using pathway databases in Cytoscape (Figures 11B,C). There are no biological processes and pathways related with cortical metabolites. Three proteins (CRAT, CPT1B, and CPT2) and two hippocampal metabolites (palmitoylcarnitine and propionylcarnitine) were involved in the fatty acid beta degradation pathway (Figure 12).

## Discussion

This study uses metabolomics to explore the candidate metabolic signatures of the cortex and the hippocampus after TBI in the subacute phase. We identified the metabolite profile for the hippocampus and for the cortex. A total of 13 differentially expressed genes (DEGs) were screened compared with the sham group. The DEGs’ function was demonstrated by KEGG analysis, which showed two metabolism-related pathways including purine metabolism for the hippocampus and taurine and hypotaurine metabolism for the cortex. Based on the HMDB and the GENECARD database, 12 of the identified proteins related to the metabolites respond to subacute TBI; seven proteins were clustered to one pathway (fatty acid beta oxidation). The results suggest that the metabolic alterations, including the identified metabolites and their enriched pathways, tend to be the potential targets of TBI diagnostics and prognosis. Furthermore, the networks of metabolite-related proteins and their interactions provide an in-depth understanding of the molecular mechanisms in the process of TBI. Additionally, demarcations between the hippocampus and the cortex following subacute TBI demonstrate that different brain regions lead to dysfunctional metabolic perturbation responses.

Recently, the hippocampus and the cortex are the key functional locations in the brain (Yin et al., 2018; Han et al., 2019; Terranova et al., 2019; Vijayakumar et al., 2019). Long-term neurodegeneration results from deficits in the cortex and the hippocampus, which induces brain damage (Arulsamy et al., 2019; Kempuraj et al., 2019). The disorders by TBI-related damage implicates chronic dysregulations with involvements of neuroinflammation (Kempuraj et al., 2019; Needham et al., 2019), immune system (Sharma et al., 2019), neuroplasticity (Yi et al., 2016) and cell survival (Hanlon et al., 2019). The roles of the hippocampus and the cortex are region-dependent alterations occurring in the brain (Yang et al., 2014; Lieblein-Boff et al., 2015; Sapiurka et al., 2016). Therefore, we identified candidate metabolites and their enriched pathways to help reveal the molecular mechanisms of TBI. Our results reveal that these metabolomic characteristics of TBI-induced effects in the hippocampus and the cortex are correlated with many metabolic disturbances.

The consequences of TBI cause changes of neuronal function, especially in the cortex (Carron et al., 2016). Increased cholecalciferol is observed in brain tissues, which is associated with the regulation of calcium homeostasis (Bivona et al., 2018), restoration of autophagy flux (Campbell and Spector, 2012; Cui et al., 2017), and decrease in apoptosis (Li et al., 2019; Molinari et al., 2019). Our results also show increased cholecalciferol in the cortex of subacute TBI, which is related to neuroprotection exertion (Mechanick et al., 1997; Van de Kerkhof et al., 2002). Differently from previous studies (Tayag et al., 1996; Liu et al., 2018), our data indicate that the levels of uric acid (UA) are significantly lower in the cortex of TBI rats. UA is a major antioxidant in the central nervous system and/or a prediction that might support good clinical recovery following TBI (Dash et al., 2016). Therefore, uric acid may play its neuroprotective role for attenuating neurological deficits. Additionally, lower betaine, trigonelline, and palmitoylcarnitine levels are detected in our results. They can regulate cellular function (Dash et al., 2016), inflammation (Farkhondeh et al., 2018; Mallah et al., 2019), and energy metabolism disorder (Ojuka et al., 2016). To date, no studies have addressed the alterations of guanidinoethyl sulfonate, ecgonine, and 2′-deoxyinosinea during TBI. Herein our results provide evidence on perturbations in the cortical metabolites of subacute TBI.

In contrast to the cortex, palmitoylcarnitine and betaine change conversely in the brain region of the hippocampus in response to subacute TBI. The evidence in previous studies suggests that betaine treatment plays a vital role in synaptic plasticity associated with learning and memory (Kunisawa et al., 2017). An increase in palmitoylcarnitine plays an important role in the decrease of fatty acid beta oxidation at the acute time point, which is consistent with our study (Ojuka et al., 2016). This overlap results suggest that the same metabolite with brain-region-dependent functions causes different alterations in brain disease. Meantime, ascorbic acid performs cerebral antioxidant effects (Prieto et al., 2011). Hypotaurine produces taurine and may be involved in age-related cognitive GABAergic system alterations in taurine and hypotaurine metabolisms (Wu et al., 2008). The hypotaurine levels are elevated in response to hypoxia to exert antioxidant protection upon reperfusion (Sakuragawa et al., 2010; Liao et al., 2018), and propionylcarnitine function is reported to be the marker of oxidative stress in the hippocampal CA1 region 7 days after ischemia (Al-Majed et al., 2006). Adenine decreases in the periods of anoxia or ischemia in myocardial ATP (Esposito et al., 1999). Overall, our findings propose hippocampal regional variations that are relevant to synaptic plasticity dysregulation and antioxidant defenses in the subacute TBI brain.

Moreover, another finding that we observed in our present results is that seven subacute hippocampal metabolites, also identified in TBI when compared with our pervious study, were related to metabolic profiles during the acute phase (Zheng et al., 2020). Our results show that these hippocampal metabolites all increased in the subacute period of TBI except L-norleucine that keeps the same level in the subacute phase when compared with that in the acute phase of TBI. According to the analysis above, the increased levels of the six metabolites, along with the same levels of L-norleucine, further illustrate the sustainability of the biological dysregulation status in the rat brain with TBI compared to the sham subjects. The increased levels of six hippocampal metabolites may be explained by the higher variations relevant to synaptic plasticity dysregulation and antioxidant defenses from the acute phase to the subacute phase in TBI. This means that a link exists between the metabolic fluctuation in TBI and the biological progression in synaptic plasticity dysregulation and antioxidant defenses before post-period in TBI. In another perspective, there is a necessity to focus on synaptic plasticity and antioxidants in the acute hippocampal TBI.

In our study, metabolites enriched in purine metabolism pathway are identified in the cortex. Purine biology developments refer to the pathomechanisms of secondary injury and the therapies for brain injury (Chaikuad and Brady, 2009; Liu et al., 2018). Our study shows altered uric acid in this pathway, which is a waste product of purine metabolism (Johnson et al., 2009). In the subacute hippocampal TBI, taurine and hypotaurine metabolism is an enriched pathway in our study. Previous metabolomics studies have reported the involvement of taurine and hypotaurine metabolism in brain diseases such as Parkinson’s disease (Shao and Le, 2019). Taurine and hypotaurine metabolism is also correlated with inflammatory responses (Shui et al., 2018). Increasing evidence in this study suggests that the metabolic changes in the pathophysiology of the cortex and the hippocampus after subacute TBI are under different states.

Our database analysis shows an accumulation of 21 different proteins and two main pathways assigned in the cortex. There are 43 different proteins and three main pathways in the hippocampus. Purine nucleoside phosphorylase may form a key enzyme in the recycling of predominantly host-derived purines and produces the major purine precursor when catalyzing the phosphorolysis of inosine (Chaikuad and Brady, 2009). Adenosine deaminase (ADA) deficiency may have a deep impact on cellular physiology by elevating the levels of ADA substrates, including adenosine and deoxyadenosine (Dolezal et al., 2005). Uric acid is the final product of xanthine dehydrogenase as well as ADA in purine metabolism in humans (Yun et al., 2017). These protein maps may be used to identify the dysfunctional metabolic enzymes when considering purine metabolism disorder in subacute cortical TBI. The action of glutamate decarboxylases (GADs) such as GAD1 and GAD2 can synthesize gamma-aminobutyric acid, which is a neurotransmitter from amino acid glutamate (Grone and Maruska, 2016). Hypotaurine could be produced involving oxidative stress and membrane damage *via* 2-aminoethanethiol dioxygenase oxidation (Liu et al., 2017). Hypotaurine can be converted by cysteine sulfinic acid decarboxylase for taurine production (Wang et al., 2018). These novel results lead to new insights of effective diagnosis and treatment.

Our protein–disease network pattern, coupled with their interaction in pathways, shows that TBI may be regulated and predicted by proteins. Fatty acid synthesis is a multistep process involving several critical enzymes (Janßen and Steinbüchel, 2014). For example, CPT1 and CPT2 mediate palmitoylcarnitine or acetyl-CoA, which are the primary and the final products of fatty acid oxidation in the mitochondria (Kerner et al., 2014; Fontaine et al., 2018; Lin et al., 2018). These results provide a global view of the interactions between metabolites and connected proteins. The revelations of metabolite–protein interactions may help to guide the treatment of TBI.

Several limitations should be addressed. We only employed LC-MS/MS technology to detect the metabolic profiles. More metabolomics technologies such as gas chromatography–mass spectrometry and NMR are needed to confirm our findings. Additionally, the sample size was relatively small. Finally, the interactions of protein expression and their related metabolites should be further investigated to broaden their therapeutic and clinical potential.

## Conclusion

In conclusion, our results represent significant profiling change and provide unique metabolite–protein information in a rat model of TBI following the subacute phase. This study may inspire scientists and doctors to further their studies and provide potential therapy targets for clinical interventions.

## Data Availability Statement

The datasets generated for this study are available on request to the corresponding author.

## Ethics Statement

All procedures involving in the experiments were approved in compliance with regulations of Medical Ethics Committee at Xiangya Hospital of Central South University.

## Author Contributions

YW, WZ, and C-SD designed the study. FZ, Y-TZ, P-FL, EH, TL, TT, and J-KL performed the experiments. FZ and TL analyzed the data. FZ visualized the figures. FZ and YW drafted the manuscript. YW, WZ, and C-SD revised the manuscript. WZ and C-SD funded the study. All authors read and approved the final manuscript.

## Conflict of Interest

The authors declare that the research was conducted in the absence of any commercial or financial relationships that could be construed as a potential conflict of interest.

## Footnotes

1 http://www.metaboanalyst.ca/

2 https://blast.ncbi.nlm.nih.gov/Blast.cgi
